# Inter- and intraobserver reproducibility of buccal bone measurements at dental implants with cone beam computed tomography in the esthetic region

**DOI:** 10.1186/s40729-015-0007-1

**Published:** 2015-04-18

**Authors:** Kirsten W Slagter, Gerry M Raghoebar, Arjan Vissink, Henny J A Meijer

**Affiliations:** 1 Department of Oral and Maxillofacial Surgery, University of Groningen, University Medical Center Groningen, PO Box 30.001, 9700RB Groningen, The Netherlands; 2 Department of Fixed and Removable Prosthodontics, University of Groningen, University Medical Center Groningen, PO Box 30.001, 9700RB Groningen, The Netherlands

**Keywords:** Dental implants, Esthetic region, CBCT, Bone thickness

## Abstract

**Background:**

Sufficient buccal bone is important for optimal esthetic results of implant treatment in the anterior region. It can be measured with cone beam computed tomography (CBCT), but background scattering and problems with standardization of the measurements are encountered. The aim was to develop a method for reliable, reproducible measurements on CBCTs.

**Methods:**

Using a new method, buccal bone thickness was measured on ten CBCTs at six positions along the implant axis. Inter- and intraobserver reproducibility was assessed by repeated measurements by two examiners.

**Results:**

Mean buccal bone thickness measured by observers 1 and 2 was 2.42 mm (sd: 0.50) and 2.41 mm (sd: 0.47), respectively. Interobserver intraclass correlation coefficient was 0.96 (95% CI 0.93 to 0.98). The mean buccal bone thickness of the first measurement and the second measurement of observer 1 was 2.42 mm (sd: 0.50) and 2.53 mm (sd: 0.49), respectively, with an intraobserver intraclass correlation coefficient of 0.93 (95% CI 0.88 to 0.96). The mean buccal bone thickness of the first measurement and the second measurement of observer 2 was 2.41 mm (sd: 0.47) and 2.52 mm (sd: 0.47), respectively, with an intraobserver intraclass correlation coefficient of 0.96 (95% CI 0.93 to 0.97).

**Conclusions:**

Applying the methods used in this study, CBCTs are suitable for reliable and reproducible measurements of buccal bone thickness at implants.

## Background

Single-tooth implant placement in the esthetic zone is a highly reliable treatment option for replacing a failing tooth [1-4]. Yet, research interest has shifted from implant survival towards optimal preservation of soft and hard tissues [5-7]. Especially in the esthetic region, buccal bone and its preservation is one of the key factors in esthetic outcome [8].

Computerized tomography (CT) scans and cone beam CTs (CBCTs) are commonly used for presurgical planning and to predict bone density and potential stability of dental implants [9]. Next to this, CTs and CBCTs also allow for measuring bone at dental implants during follow-up [10,11]. The quality and accuracy of a three-dimensional (3D) model derived from a (CB)CT is dependent on scanner-related factors such as type of scanner, field of view (FoV), artifacts, and voxel size [12]. In addition, patient-related factors such as patient position and metal artifacts [13] and operator-related factors such as the segmentation process or interpretation of the (CB)CT are of influence [14]. It has been reported that buccal bone thickness at implant sites can be measured with CBCT, but background scattering and problems with standardization of the measurements are frequently encountered [15]. In view of the aforementioned factors, there is need for a reliable, reproducible method to facilitate measurements. The use of 3D image diagnostic and treatment planning software programs in combination with software programs for tracking and registration of the exact position of existing dental implants in radiographs can be of help [16].

The aim of the current study was to develop a reproducible method based on 3D image diagnostic and treatment planning software programs for buccal bone measurements at implants on CBCTs.

## Methods

**Figure 1:**
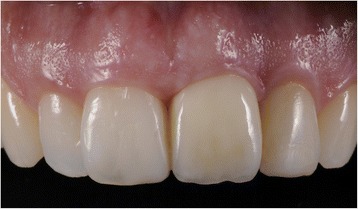
**Clinical photograph of implant-supported restoration at position 21.**

**Figure 2:**
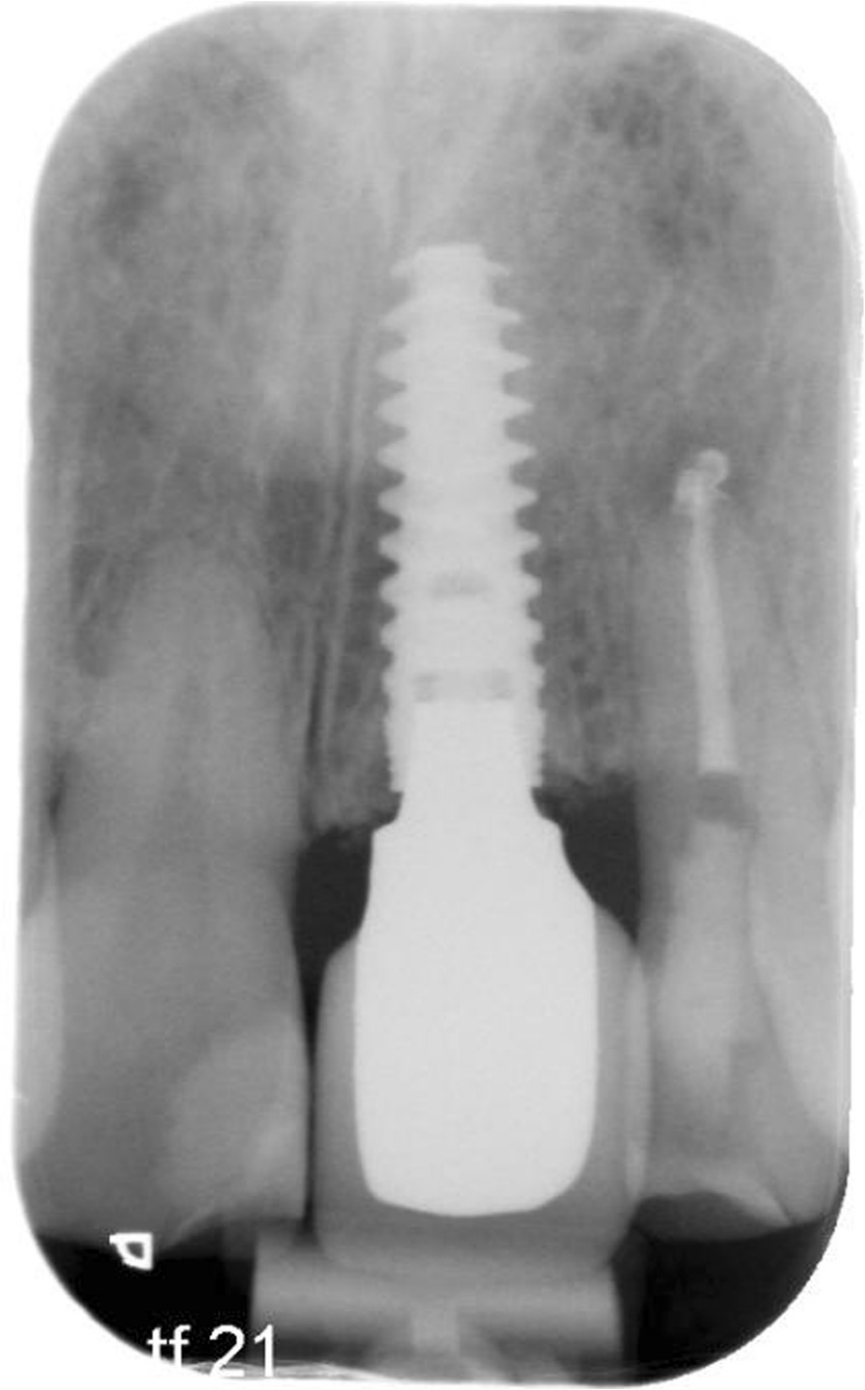
**Conventional intra-oral radiograph of same patient with implant-supported restoration at position 21.**

Ten patients with a dental implant in the esthetic zone (regions 13 to 23) were included (Figures 1 and 2). Research was carried out in compliance with the Helsinki Declaration. Patients were part of a randomized controlled trial on esthetics; the study was approved by the Medical Ethic Board of the University Medical Center Groningen, University of Groningen (METC 2010.246) as well as that written informed consent was obtained from all patients. The CBCT scans were made with an iCAT 3D exam scanner (KaVo Dental GmbH, Biberach, Germany), which scanner was validated for measuring bone thickness by Fourie et al. [17]. The method error of this scanner is very small, i.e., 0.05 mm (95% CI 0.03 to 0.07). The standard used voxel size was 0.30 and FoV was 100 × 100 mm on the CBCT scans. Bone measurements at implants on the CBCT scans was done using 3D image diagnostic and treatment planning software (NobelClinician, version 2.1 (Nobel Biocare - Guided Surgery Center, Mechelen, Belgium). A novelty is that this program, regularly used preoperatively, was employed to measure the buccal bone thickness (in mm), after implant surgery. To allow for reproducible measurements, a CBCT imaging and software protocol was developed.

### CBCT imaging and software protocol

**Figure 3:**
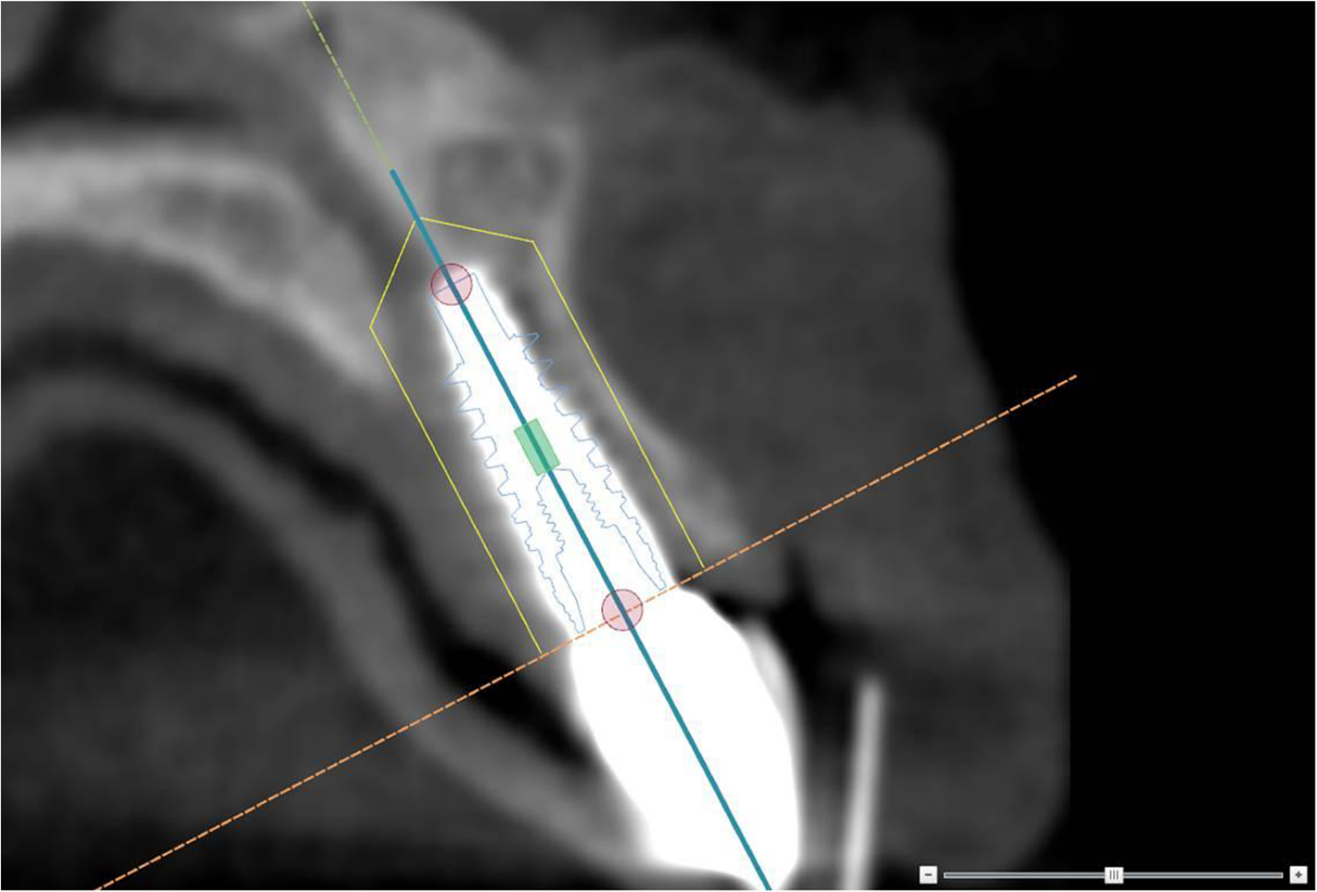
**Implant position.** Due to the alienation of the patients’ DICOM files by MIRIT, the exact position of the implant was defined. As such, the measurements could take place in the exact correct buccal direction.

Acquired CBCT Digital Imaging and Communications in Medicine (DICOM) datasets were transferred to a computer. The CBCT images were exported in DICOM multi-file format and imported into Maxilim, version 2.3 (Medicim, Sint-Niklass, Belgium). Maxilim is a medical image computing program assessing the patients head anatomy and is used for diagnostics and preoperative planning of maxillofacial surgery. The input information for Maxilim is a 3D dataset, often (CB)CT data. The DICOM files of all patients were set continuously on Hounsfield unit (HU) isovalue 280. The implant used was set on HU isovalue 130. With Multimodality Image Registration using Information Theory (MIRIT), which has an accuracy of a subvoxel, the exact position of the implant could be recognized, determined and implemented in the patients DICOM files [16]. The MIRIT procedure is based on recognizing image similarities. The degree of similarity between intensity patterns in two images is determined, and consequently, the recognized image is registered automatically into one coordinate system. Image similarities are broadly used in medical imaging to enhance diagnostics. In the software program NobelClinician, the patients DICOM files were opened with the same HU isovalue of 280. An extra research tool was added to this software program by the program makers, so that the DICOM file from Maxilim was recognized by this program and the exact position of the implant, as determined in Maxilim, could be aligned with a planning implant in NobelClinician. Due to the alignment of a planning implant (with a known configuration) and an actual inserted implant into one image, measurements could take place at the exact buccal midline of the implant (Figure 3). The display of the implant and surrounding structures was set on bone value, so that the outline of the bony structures could be seen and measured. The buccal bone measurements at midline of the implant were performed with the standard provided measurement tools in the software program of NobelClinician.

### Measuring procedure

**Figure 4:**
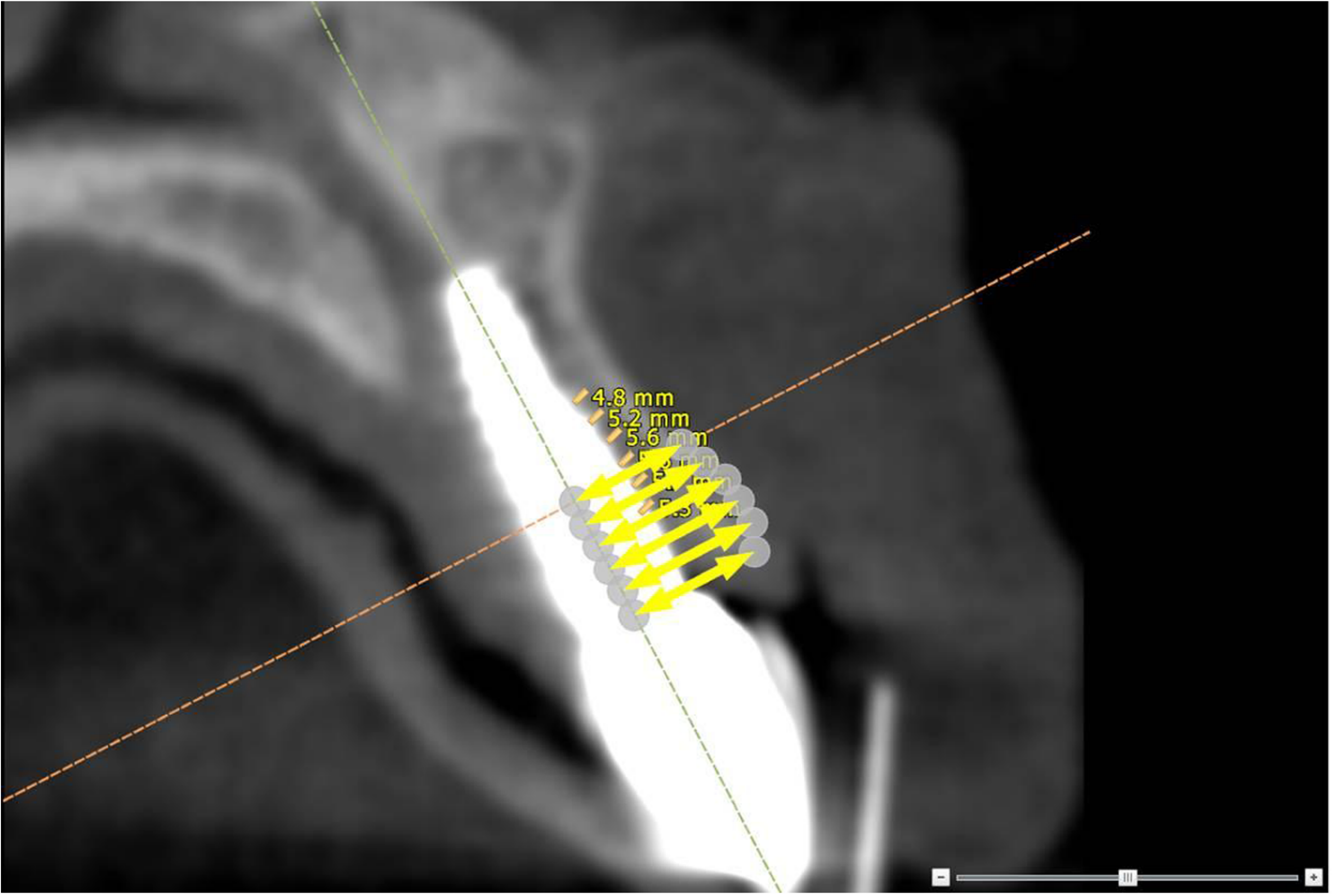
**Implant measurements.** Measurements were performed at each millimeter along the axis of the implant for 5 mm, beginning at the neck of the implant.

**Figure 5:**
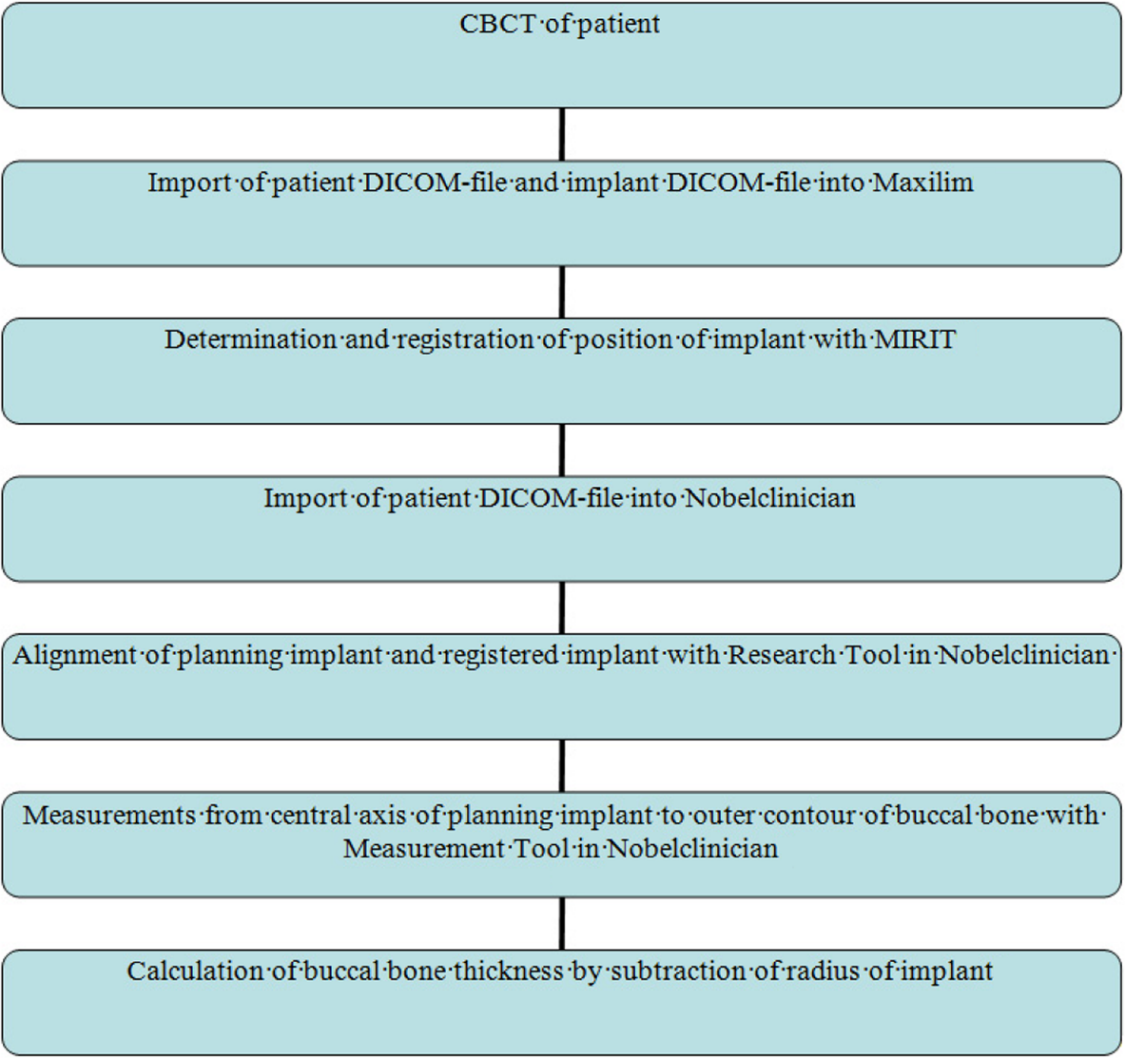
**Flow diagram of CBCT imaging and measurements to calculate bone thickness buccally of implants.**

The implant and patient dataset were exactly aligned by the MIRIT method, so that the distance from the central axis of the implant to the outer contour of the buccal bone could be measured. Area of interest was the upper 5 mm section of the implant, beginning at the neck of the implant towards the apical direction. Exact dimensions along the implant axis of each implant configuration used in the study were provided by the manufacturer. Buccal bone measurements (in mm) were performed calculating the distance to the buccal bone outline minus the radius of the interior contour of the implant. These buccal bone measurements were done for 5 mm at each millimeter along the axis, beginning at the neck of the implant (Figure 4). Measurements were repeated twice (with time interval to prevent recollection) by two independent operators (HJAM and KWS, both dentists) in a random order. Flow diagram of the consecutive steps has been depicted in Figure 5.

### Statistical analysis

Continuous variables were expressed as a mean with standard deviation. Interobserver and intraobserver variability was assessed using two-way mixed intraclass correlation coefficient single measures analysis [18]. All analyses were performed using SPSS software (version 20.0).

## Results

The mean buccal bone thickness measured by observers 1 and 2 was 2.42 mm (sd: 0.50) and 2.41 mm (sd: 0.47), respectively. Interobserver intraclass correlation coefficient was 0.96 (95% CI 0.93 to 0.98). The mean buccal bone thickness of the first measurement and the second measurement of observer 1 was 2.42 mm (sd: 0.50) and 2.53 mm (sd: 0.49), respectively, with an intraobserver intraclass correlation coefficient of 0.93 (95% CI 0.88 to 0.96). The mean buccal bone thickness of the first measurement and the second measurement of observer two was 2.41 mm (sd: 0.47) and 2.52 mm (sd: 0.47), respectively, with an intraobserver intraclass correlation coefficient of 0.96 (95% CI 0.93 to 0.97).

## Discussion

Intraobserver and interobserver agreement was very high with measurements on CBCTs of bone buccally of dental implants. Apparently, the method is clear and measurements can be performed reproducibly. Moreover, measurements are not observer dependent, meaning that results of different observers in different studies can be compared with each other.

In previous studies, buccal bone thickness was also measured, but the exact position of these measurements at the surface of the implant was not determined by 3D image-based diagnostic and treatment planning software programs [10,11,15]. It is important to perform measurements of bone thickness at the same position at implants to make comparison in time possible. Because of the cylindrical contour of the implant, thickness of bone can vary considerably in the mesio-distal direction. The combination of the software programs MIRIT (for determination and registration of the implant position in Maxilim) and Research Tool in NobelClinician (for alignment of planning implant and registered implant) makes the method reproducible.

Scattering of the titanium dental implant makes it difficult to perform measurements from the bone-to-implant boundary to the buccal outer contour of the bone [19]. The combination of Research Tool in NobelClinician (exact positioning of the planning implant) and Measurement Tool in NobelClinician (for measurements from central axis of the implant) makes it possible to bypass the scattering area. Measurements are corrected by subtraction of the known radius of implant, resulting in the actual thickness of bone.

Measurements are not directly possible in NobelClinician, because the image-recognizing program MIRIT can only be executed in the configuration of Maxilim. It would be desirable if the total procedure could be carried in one program, being NobelClinician.

## Conclusions

When applying 3D image-based software programs according to the set-up used in this study, CBCTs are suitable for reliable and reproducible measurements of buccal bone thickness at implants.

## Abbreviations

3D: three-dimensional
CBCT: cone beam computed tomography
CT: computerized tomography
DICOM: Digital Imaging and Communications in Medicine
FoV: field of view
HU: Hounsfield unit
MIRIT: Multimodality Image Registration using Information Theory
